# The International Medical Congress of 1887

**Published:** 1885-08

**Authors:** 


### EDITORIAL.

THE INTERNATIONAL MEDICAL CONGRESS OF 1887.

It is generally, if not universally, conceded that the prospect of having a successful congress of the medical profession in Washington in 1887 has been materially impaired.

At least three causes have contributed to this result.

The first, probably, rests with the original Committee of Arrangements, that did much of the work assigned to it remarkably well, when it is considered how great and how varied that work proved to be.

The original seven members of that committee, with the President of the American Medical Association added, making eight, were authorized by the American Medical Association when their appointment was made, to add to their number, in case the invitation which they were delegated to extend to the International Medical Congress that was about to convene in Copenhagen, should be accepted. The committee acted upon the authority which had been delegated to it, and added the names of many able and representative men in different sections of our country to the committee. Thus enlarged, the committee proceeded to make a preliminary schedule, which embraced the names of physicians eminent in the different departments to which they were assigned, many of that number having international reputations. Most, if not all, assigned to these positions accepted them, and began preparations for the work for which they were chosen. The committee then issued the schedule, which it had arranged, just before the last annual meeting of the American Medical Association. As this Association had been recognized by the medical profession of America as its representative body, and it had been requested by eminent members of the profession of our country to ask, *on behalf of the medical profession of America*, that the next triennial International Medical Congress might be held in Washington, it was not unnatural that the Association expected that the Committee of Arrangements, that it had empowered to act, and for whose acts as its appointees it became in a measure responsible to the profession at large, to report to it, officially, the acceptance of the invitation that had been given, It was right that a report should be made to it of the work which had already been done by the committee, for its approval or modification, before distributing the schedule at home and abroad. No doubt that distribution was made with the best of intent, and in the belief of its correctness, but that it proved to be unfortunate and impolitic is more than likely.

Another mistake is believed to have been made by that committee in so limiting the number, in their additions to the original eight, that several important official positions had to be assigned to single individuals, when it is claimed that additional and equally eligible and equally capable men in the profession might have been added to the committee and assigned to some of the multiple offices given to a relatively large proportion of residents of the Atlantic slope of the Alleghanies. Whilst geographical considerations alone should not decide selections for representatives in a scientific body, yet as the invitation was that of the whole profession of America, the American Medical Association having only been asked to become sponsor for it, it was but right that due regard should be had to locality after professional qualifications had been, first, duly considered. It is not improbable that more liberality of views in this respect on the part of a portion of the committee, would have secured more harmony and more cordial coöperation. However, the fact should be kept prominent that the contemplated congress is designed to be, as all its predecessors have been, international in its broadest sense. Although the medical profession of America and, as representing it, the American Medical Association, stand as sponsors for the next congress, and, in a certain sense, as host for the foreign members who have been invited, yet all the arrangements should be catholic, in the best sense of that term, and that in no sense can the congress be, nor should it be, controlled either by the American Medical Association or by the medical profession of America.

The next mistake was made by the American Medical Association at its meeting in New Orleans. Whilst granting to it the right to review, to criticize and even to amend the acts of the committee that it had created and empowered to act for it, and granting still further that it had a right to add to its committee, it is not clear on what grounds it ignored the right of the original committee of eight to add to its number, after having distinctly authorized such addition at its annual meeting the year before. It did not claim that the members added by the committee of eight were not capable and representative men, but it made the unfortunate and impolitic mistake of introducing a local ethical question into an international scientific body, that ignores all such questions, and places science and philanthropy above and beyond ethical creeds.

This led to the result that able and representative men were deposed, and even if their places have been filled by equally capable men, the effect upon the prospects of the congress has been very serious. It enlarged its committee of arrangements—a privilege that, it is conceded, it had a right to exercise—but at the same time it eliminated from that committee those members whom the original committee of eight had, by the authority conferred upon it, added to its number, and who thereby became as much a part of the committee of arrangements as the original eight members themselves. This was unnecessary, unjust, and wrong, and it is but charitable to suppose that in the haste and the heat of discussion, the wrong was, unintentionally and without due thought, committed. If so, the mistake should be corrected by inviting those so excluded to resume their places with the other members of the committee, which should then consist of the original eight, the members that those eight added to their number, and the additional members which the association selected at New Orleans. It is true that some of the original eight have resigned the task they had undertaken. Under all the circumstances it may not have been unnatural that they should have done so, viewing matters from the stand-point of self; but in view of the fact that they presented the invitation that was accepted ; that they pledged the best efforts of the profession of America to make a success of the next congress, from a professional, a scientific and a social stand-point, they should not have been among the first to abandon their posts. They should be invited to resume their places on the committee, and they should accede to that request The question is no longer one of personal preference or personal taste. Our faith as a profession has been pledged, and we are in honor bound to make good our pledge. We have a right to expect that those who were foremost in asking the medical profession of the world to come to our shores will not be foremost in abandoning their posts of trust, because others, as well as themselves, have made mistakes. That some unfortunate selections have been made by the American Medical Association is apparent, but it is as true as ever, that two wrongs never make one right.

The third contributing cause seems to lie with the committee of arrangements, as at present constituted.

The persistency with which, it is alleged, it thrusts the question of local professional ethics forward, is alienating the foreign members of our profession whom we have hoped to see among us, but who are declaring that they see, in this most injudicious wrangling, evident signs of failure of the congress, unless this discord should end and the course be adhered to which has characterized each congress that has so far been held.

Although but one meeting of that committee has been held, and the details of its future plans have not been fully announced, yet such intimations have gone forth as to have had a very unfortunate effect upon a large proportion of prominent men in the profession in our country, who by their hasty and, perhaps, injudicious course are further embarrassing an honest effort on the part ofthe mass of the profession to make good the faith pledged at Copenhagen, and to save the good name of our country from the disgrace, and to avert the obloquy which failure to meet the expectations that we have raised abroad must bring. It seems incomprehensible that men, who have fairly won distinction in the profession of medicine, should be found banding themselves together in a manner suggestive of trades-unions, and placing impediments in the way of making a success of a scientific and philanthropic congress. Most careful sifting for reasons for such course fails to discover other than the fact that the American Medical Association has exercised a right, that it clearly possessed, to revise the work of its committee; that it did it in an obnoxious way; that it thrust and, through its committee, it continues to thrust, a question of local professional ethics into a scientific body, to its great detriment, and that it has placed in prominent official positions a few men whom the profession of the country are unwilling to regard as representative, and who are obnoxious to, at least, one of the factions in this unseemly wrangle.

If, for argument's sake, it be granted that these positions were well taken, does it follow that the course taken by these disaffected members of the profession is justifiable or that it will bear reflection. Can they, for such reasons, afford to invite disaster in a cause in which the good name of the medical profession of our country is involved? Would it not be much more to their credit that they should be found in the ranks, striving to preserve our name untarnished, rather than to be surrendering the field, at the first skirmish, to those whom they are unwilling to acknowledge as their peers?

The only practicable solution of this most unfortunate dilemma seems to lie in an abandonment of a rule-or-ruin policy, and recognition of the mistakes made. This would prepare a way for honorable compromise and bringing together all who have been appointed to arrange the preliminaries essential to the proper opening of the Congress.

Good faith requires that an honest effort should be made to do the best that is possible, under these embarrassing circumstances, to make a success of the proposed congress. If failure and disgrace result, let the responsibility rest where it properly belongs, and let it be admitted that American physicians showed their lack of capacity to make good their promise, and to organize a congress, after having repeatedly expressed a desire to have their country honored by selecting it as a place for its meeting, and after having extended a formal invitation and having had it accepted.

Then let the shame be confessed, and, in fitting acknowledgement of their unworthiness to participate, let Americans, thereafter, absent themselves from every international medical congress that may be held.

